# Unique Mechanism of the Interaction between Honey Bee Toxin TPN_Q_ and rKir1.1 Potassium Channel Explored by Computational Simulations: Insights into the Relative Insensitivity of Channel towards Animal Toxins

**DOI:** 10.1371/journal.pone.0067213

**Published:** 2013-07-10

**Authors:** Jun Hu, Su Qiu, Fan Yang, Zhijian Cao, Wenxin Li, Yingliang Wu

**Affiliations:** State Key Laboratory of Virology, College of Life Sciences, Wuhan University, Wuhan, P. R. China; Instituto de Tecnologica Química e Biológica, UNL, Portugal

## Abstract

**Background:**

The 21-residue compact tertiapin-Q (TPN_Q_) toxin, a derivative of honey bee toxin tertiapin (TPN), is a potent blocker of inward-rectifier K^+^ channel subtype, rat Kir1.1 (rKir1.1) channel, and their interaction mechanism remains unclear.

**Principal Findings:**

Based on the flexible feature of potassium channel turrets, a good starting rKir1.1 channel structure was modeled for the accessibility of rKir1.1 channel turrets to TPN_Q_ toxin. In combination with experimental alanine scanning mutagenesis data, computational approaches were further used to obtain a reasonable TPN_Q_ toxin-rKir1.1 channel complex structure, which was completely different from the known binding modes between animal toxins and potassium channels. TPN_Q_ toxin mainly adopted its helical domain as the channel-interacting surface together with His12 as the pore-blocking residue. The important Gln13 residue mainly contacted channel residues near the selectivity filter, and Lys20 residue was surrounded by a polar “groove” formed by Arg118, Thr119, Glu123, and Asn124 in the channel turret. On the other hand, four turrets of rKir1.1 channel gathered to form a narrow pore entryway for TPN_Q_ toxin recognition. The Phe146 and Phe148 residues in the channel pore region formed strong hydrophobic protrusions, and produced dominant nonpolar interactions with toxin residues. These specific structure features of rKir1.1 channel vestibule well matched the binding of potent TPN_Q_ toxin, and likely restricted the binding of the classical animal toxins.

**Conclusions/Significance:**

The TPN_Q_ toxin-rKir1.1 channel complex structure not only revealed their unique interaction mechanism, but also would highlight the diverse animal toxin-potassium channel interactions, and elucidate the relative insensitivity of rKir1.1 channel towards animal toxins.

## Introduction

The diverse and ubiquitous potassium channels serve a variety of physiological and pharmacological functions [1]. These proteins are often targeted by numerous peptide toxins from the venomous animals, such as scorpions, spiders, sea anemones, honey bees, snakes and cone snails [2]. Nowadays, the structural interactions between potassium channels and animal toxins are an intense research field due to the following two advantages. First, more structural information on the potassium channel-animal toxin interactions can be obtained. Using computational techniques in combination with the experimental data, many potassium channel-animal toxin complex structures were predicted, such as *shaker* channel-*κ*-PVIIA toxin complex [3], hERG channel-BeKm-1 toxin complex [4], Kv1.1 channel-ADWX-1 toxin complex [5]; BK_Ca_ channel-ChTX toxin complex [6], Kv1.3 channel-Hg1 toxin complex [7]. These progresses not only indicated the diverse structural information on the potassium channel-animal toxin interactions, but also provided various dynamic structure features of potassium channels induced by toxin recognition. Second, the screening and design of toxin peptide drugs can be accelerated. With more potassium channels as the therapeutic targets [8], the rational screening and design of peptide drugs, based on the structural information on the potassium channel-animal toxin interactions, exhibited an attractive prospect for disease diagnosis and treatment [9]–[12]. Facing the fact that crystallization and determination of potassium channel-animal toxin complex structures remain a huge challenge, the computational approaches are greatly essential to study the interactions of animal toxins with the potassium channels.

TPN_Q_ toxin is a derivative of 21-residue honey bee toxin Tertiapin (TPN), whose Met13 residue is substituted by a glutamine residue [13]. Different from the classical structures of scorpion toxins acting on the potassium channels, TPN_Q_ toxin has only an α helix without β sheet domains, whose different structural parts are held together by two pairs of disulfide bonds. TPN_Q_ can inhibit rKir1.1 channel with a K_d_ value of 1.3 nM [13]. By using alanine-scanning mutagenesis, the binding interface of TPN_Q_ toxin was primarily formed by its α helical domain, indicating that TPN_Q_ toxin would adopt a novel mechanism to recognize rKir1.1 channel [14]. Meanwhile, rKir1.1 channel, with only two transmembrane helices and a pore domain, was found to use its vestibule to associate with TPN_Q_ peptide from the alanine-scanning mutagenesis data [14]. According to the crystal structure of homologous chicken Kir2.2 (cKir2.2) channel [15], rKir1.1 channel has two unique structural features in its outer vestibule: (1) the four turrets are likely larger and come closer together, constricting the pore entryway compared to the classical Kv1.2 channel [16]; (2) two strong hydrophobic Phe146 and Phe148 residues locate near the selectivity filter while four Phe146 residues possibly form protrusions on the surface at the pore region. These structural features suggest that rKir1.1 channel likely use a novel mechanism to interact with TPN_Q_ peptide. To further characterize the novel interaction of toxin TPN_Q_ with rKir1.1 channel at the structural level, the computational approaches were used to simulate the reasonable TPN_Q_ toxin-rKir1.1 channel complex structure in this work. On the basis of this complex structure, the unique molecular mechanism of the interaction between TPN_Q_ toxin and rKir1.1 channel was elucidated. These findings were helpful to highlight the diversity of animal toxin-potassium channel interactions, and elucidate the relative insensitivity of rKir1.1 channel towards animal toxins.

## Materials and Methods

### Atomic coordinates and molecular docking

Amino acid sequences of the rKir1.1 and cKir2.2 channels are obtained from the National Center for Biotechnology Information (NCBI) protein database (NCBI entries P35560.1 and XP_425235.2, respectively). The spatial structure of rKir1.1 channel was modeled by using the crystal structure of the cKir2.2 channel from the Protein Data Bank (PDB) (PDB code: 3JYC) [15], [17] as a template through the SWISS-MODEL server [18]. Our previous segment-assembly homology modeling method [4], [6] was used to refine the modeled rKir1.1 structure. The amino acid sequence of TPN_Q_ toxin is “ALCNCNRIIIPHQCWKKCGKK”. The homologous structures of TPN_Q_ toxin were modeled by using the atomic coordinates of TPN structures (PDB code: 1TER) [17], [19].

The structures of TPN_Q_ toxin were used to dock with the rKir1.1 structure through the ZDOCK program, a Fast Fourier Transform (FFT)-based, initial-stage rigid-body molecular-docking algorithm [20]. Clustering analysis and experimental data-based screening [14] were then carried out on all the complexes to select the possible hits, appropriate candidate complexes were identified for further molecular dynamic simulation study.

### Molecular dynamics simulations

In this work, all the Molecular Dynamic (MD) simulations were performed using the Amber 11 program [21] on a 128-CPU Dawning TC5000 cluster (Beijing, China). The ff99 force field (Parm 99) [22] was applied throughout all the simulation steps. As the animal toxin inhibitors bind to the extracellular part of potassium channels, where the interaction between the toxins and potassium channels might be less affected by the membrane environment, the membrane around the potassium channels sometimes is not considered during the simulation for facilitating the computations [4]–[6], [9], [24]. In this work, the membrane around the transmembrane helices of rKir1.1 channel was not used during the MD simulations since TPN_Q_ toxin was found to bind to the extracellular part of rKir1.1 channel according to the mutagenesis studies [14].

The screened TPN_Q_ toxin-rKir1.1 channel complex structures were subjected to unrestrained simulations in explicit solvent systems. They were embedded in a periodic water box, and were then subjected to 1.5 ns equilibration and 10 ns unrestrained simulations by using the sander module in Amber11 program [21]. The equilibration steps were taken by gradually reducing the force constant from 5.0 (kcal/mol)/Å^2^ for restraining all the heavy atoms to 0.02 (kcal/mol)/ Å^2^ for backbone heavy atoms only. The temperature was set at 300 K with a cutoff distance of 10 Å.

### Calculation of binding free energy

In the Molecular Mechanics–Generalized Born Surface Area (MM-GBSA) method of AMBER 11 [21], the binding free energy of 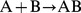 is calculated using the following thermodynamic cycle:
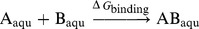


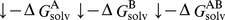


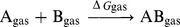


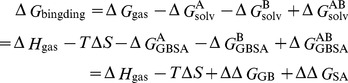
(1)

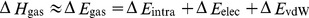
(2)

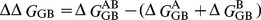
(3)

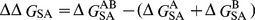
(4)Where *T* is the temperature, *S* is the solute entropy, 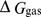 is the interaction energy between A and B in the gas phase, and 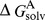, 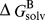, and 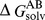 are the solvation free energies of A, B, and AB, which are estimated using the GBSA method [21]. That is, 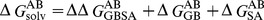, and so forth. 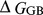 and 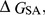 are the electrostatic and nonpolar term, respectively. 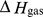 is the enthalpy in the gas phase. 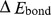, 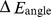, and 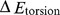 are contributions to the intramolecular energy 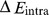 of the complex. 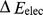 is electrostatic (elec) energy, and 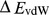 is van der Waals (vdW) interaction energy. Because of the constant contribution of 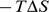 for each docked complex, we quote 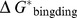 for 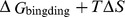 in the discussion. To verify the quality and validity of the resulting TPN_Q_ toxin-rKir1.1 channel complexes, the relative binding free energy 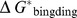 was calculated by using MM-GBSA method for postprocessing collected snapshots from the MD trajectories, and the main parameters were used as following: The IGB value was 2 for activating the Onufriev's GB parameters; the SURFTEN value was 0.0072 for computing the nonpolar solvation free energy with the LCPO method; the SALTCON value of 0.1 M was given as the concentration of mobile counterions in solution; the EXTDIEL value of 80.0 was used as the dielectric constant for the solvent, and the INTDIEL value of 1.0 was set as the dielectric constant for the solute.

## Results

### Structural modeling and refinement of rKir1.1 channel

**Figure 1:**
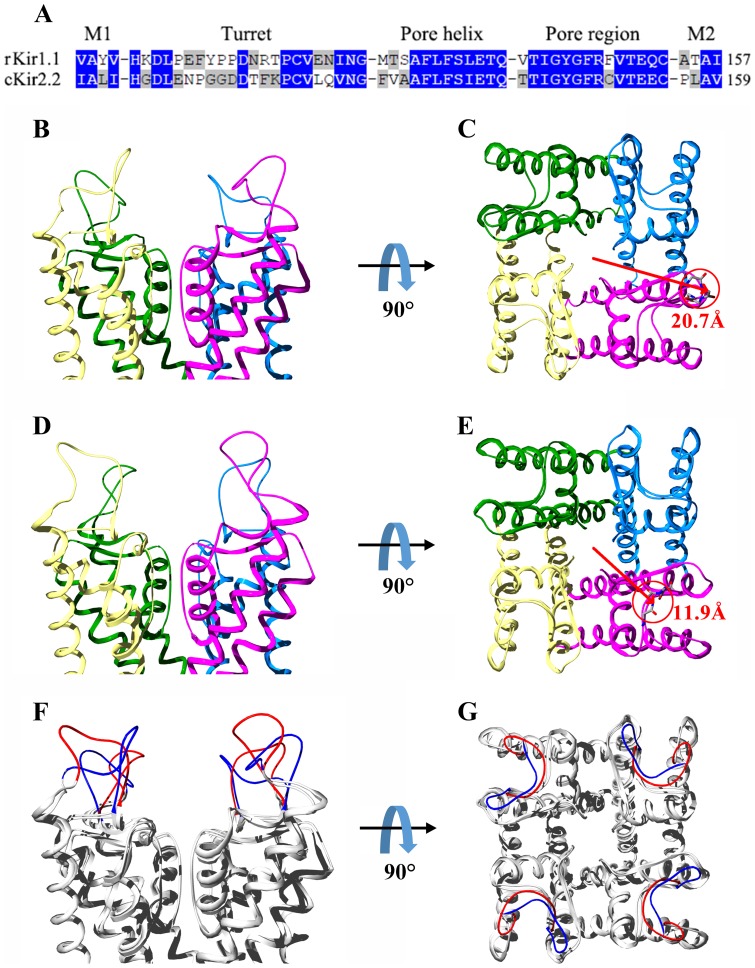
Sequence alignment and view of modeled rKir1.1 channels. (A) Sequence alignment between rKir1.1 and cKir2.2 channels. The conserved residues are blue-shaded, and the variable residues are gray-shaded. (B and C) The side and top view of the modeled structure of rKir1.1 channel by using cKir2.2 channel as the template. The distance between the C_α_ atom of Asn117 residue and the channel pore central axis was 20.7 Å. (D and E) The side and top view of the refined structure of rKir1.1 channel. The distance between the C_α_ atom of Asn117 residue and the channel pore central axis was 11.9 Å. (F and G) The side and top view of the modeled and refined structures of rKir1.1 channel. The loop segment (residue 110–120) of the modeled channel turret is blue-colored, and the counterpart of the refined channel turret is red-colored.

The starting structure of rKir1.1 channel is essential for investigating its interaction with TPN_Q_ toxin. Based on the 54.63% sequence identity between rKir1.1 and cKir2.2 channels (Fig. 1A), the structure of rKir1.1 channel was first modeled by using cKir2.2 channel structure as the template [15]. As shown in Fig. 1B and 1C, the four turrets in rKir1.1 channel structure resembled those of cKir2.2 channel, and formed a narrower pore entry compared to the classical Kv1.2 channel [16]. Previous mutagenesis data showed that rKir1.1 channel turret formed the binding site for TPN_Q_ toxin [14]. In the rKir1.1 channel structure, these functional residues in the channel turrets, such as Asp116, Asn117, Arg118 and Thr119 residues responsible for TPN_Q_ toxin binding [14], were found far away from the docked TPN_Q_ toxin. The distance between the C_α_ atom of Asn117 residue in the turret and the channel pore central axis was about 20.7 Å so that the 21-residue TPN_Q_ toxin with small size could not contact with the functional residues in channel turrets within a distance of 5 Å in the predicted TPN_Q_ toxin-rKir1.1 channel complexes (data not shown) (Fig. 1C). These disassociations between TPN_Q_ toxin and rKir1.1 channel turrets did not change even if the TPN_Q_ toxin-rKir1.1 channel complexes were subjected to 5 ns unrestrained MD simulations according to our previous work [9], [23]. These information suggested that the modeled rKir1.1 channel structure was necessary to be further refined for TPN_Q_ toxin docking experiments.

Our previous work indicated that the turret conformation of potassium channels was flexible induced by animal toxin binding [4]–[6], [23]. Here, we remodeled the turret structure of rKir1.1 channel by our previous segment-assembly homology modeling method [4], [6]. In the refined rKir1.1 channel structure (Fig. 1D and 1E), the distance between the C_α_ atom of Asn117 residue and the channel pore central axis was 11.9 Å, which was much shorter than that of previous rKir1.1 channel structure (Fig. 1C). More importantly, the functional residues Asp116, Asn117, Arg118 and Thr119 could contact TPN_Q_ toxin within a distance of 5 Å in the predicted TPN_Q_ toxin-rKir1.1 channel complexes. By comparing the two rKir1.1 channel structures, the significant conformational differences located in the loop segment (residue 110–120) of channel turret (Fig. 1F and 1G). This refined rKir1.1 channel structure was used for TPN_Q_ toxin docking, and the following reasonable TPN_Q_ toxin-rKir1.1 channel complex structure indicated the conformational flexibility of rKir1.1 channel turret.

### Discrimination of plausible binding modes

**Figure 2:**
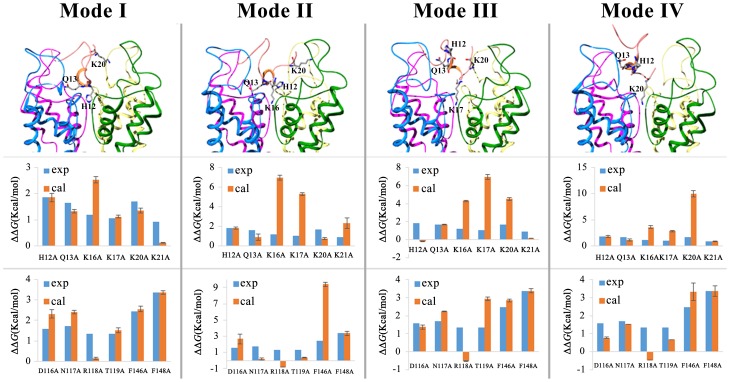
Structural view of four TPN_Q_-rKir1.1 binding modes with comparison of calculated and experimental effects. Each row of figures is as follows: (top) the differential spatial orientations of TPN_Q_ toxin in complex with rKir1.1 channel with His12, Lys16, Lys17 and Lys20 as pore-blocking residue, respectively. The most critical residues His12, Gln13 and Lys20 of TPN_Q_ toxin are labeled. (middle) The comparison of calculated and experimental effects for the six alanine mutations of TPN_Q_ toxin on the binding affinity towards rKir1.1 channel after 2.5 ns MD simulations. The calculated results are normalized values of ΔΔ*G*_binding_, whereas experimental results are obtained as *k_b_T* ln [IC_50_(mutant)/IC_50_(wt)]. (bottom) The comparison of calculated and experimental effects for the six alanine mutations of rKir1.1 channel on the binding affinity towards TPN_Q_ toxin after 2.5 ns MD simulations. The calculated results are normalized values of ΔΔ*G*_binding_, whereas experimental results are obtained as *k_b_T* ln [IC_50_(mutant)/IC_50_(wt)].

Based on the refined rKir1.1 channel structure, the candidate TPN_Q_ toxin-rKir1.1 channel complex structures were predicted by ZDOCK program [20]. Through clustering analysis, four main binding modes were found according to the pore-blocking residues of TPN_Q_ toxin: (1) Mode I: His12 as pore-blocking residue; (2) Mode II: Lys16 as pore-blocking residue; (3) Mode III: Lys17 as pore-blocking residue; (4) Mode IV: Lys20 as pore-blocking residue (Fig. 2, top panel). In order to find more reasonable binding mode, the representative candidate TPN_Q_ toxin-rKir1.1 channel complex structures from four binding modes were subjected to energy minimization, successively followed by 1.5 ns restrained MD simulations and 1 ns unrestrained MD simulations. Then the computational alanine scanning method in MM-GBSA was used to calculate the ΔΔ*G*_binding_ values for 6 main single mutations of TPN_Q_ toxin (Fig. 2, middle panel) and 6 main single mutations of rKir1.1 channel (Fig. 2, bottom panel). Overall, it was easy to find that the binding mode I was in accordance with experimental data from the alanine scanning mutagenesis studies [14], and other three binding modes showed various disagreements between the computational and experimental data. For example, the ΔΔ*G*_binding_ values of both K16A and K17A were much bigger than experimental data in other three binding modes, and the ΔΔ*G*_binding_ value of H12A was negative in the binding mode III while His12 residue seriously affected TPN_Q_ toxin binding affinity [14]. Together, the TPN_Q_ toxin-rKir1.1 channel complex structure from the binding mode I was selected for further computation and analysis.

### Validity of TPN_Q_ toxin-rKir1.1 channel complex

**Figure 3:**
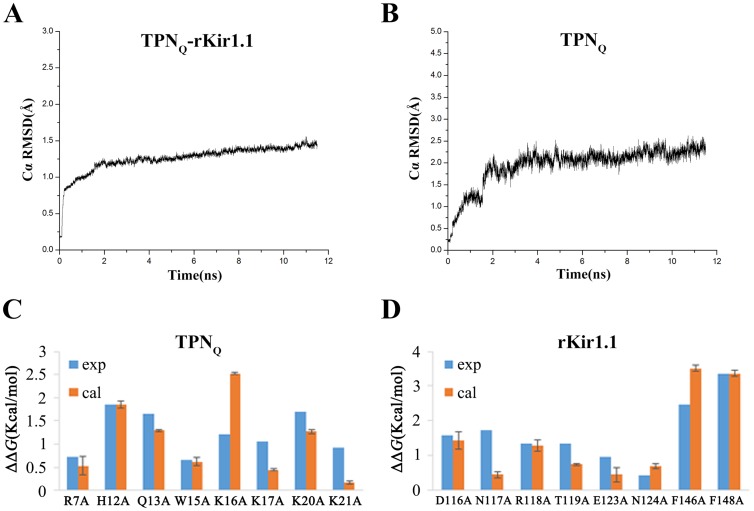
The stability and validity of the final TPN_Q_-rKir1.1 complex. (A) RMSD of the C_α_ atom in the final TPN_Q_-rKir1.1 complex from the starting complex during the 1.5 ns restrained MD simulations and 10 ns unrestrained MD simulations. (B) RMSD of the C_α_ atom of TPN_Q_ toxin from the starting complex during the 1.5 ns restrained MD simulations and 10 ns unrestrained MD simulations. (C) The comparison of calculated and experimental effects for the eight alanine mutations of TPN_Q_ toxin on the binding affinity towards rKir1.1 channel after 1.5 ns restrained MD simulations and 10 ns unrestrained MD simulations. The calculated results are normalized values of ΔΔ*G*_binding_, whereas experimental results are obtained as *k_b_T* ln [IC_50_(mutant)/IC_50_(wt)]. (D) The comparison of calculated and experimental effects for the eight alanine mutations of rKir1.1 channel on the binding affinity towards TPN_Q_ toxin after 1.5 ns restrained MD simulations and 10 ns unrestrained MD simulations. The calculated results are normalized values of ΔΔ*G*_binding_, whereas experimental results are obtained as *k_b_T* ln [IC_50_(mutant)/IC_50_(wt)].

To make the selected complex more stable and reliable, a further 9 ns unrestrained MD simulations were performed to enough equilibrate the TPN_Q_ toxin-rKir1.1 channel complex structure. As shown in Fig. 3A and 3B, the little variance of C_α_ atom Root-Mean-Square Deviation (RMSD) for both complex and TPN_Q_ toxin was found at the end of the simulations, which indicated that the system was sufficiently equilibrated.

On the basis of the equilibrated TPN_Q_ toxin-rKir1.1 channel structure, its validity was further investigated by the computational alanine scanning method. The ΔΔ*G*_binding_ values of 8 single mutations of TPN_Q_ toxin and 8 single mutations of rKir1.1 channel were calculated and compared with the experimental data, respectively [14]. As shown in Fig. 3C and 3D, an overall high degree of correlation was found between the computational and experimental data. For TPN_Q_ toxin, the big calculated ΔΔ*G*_binding_ values of H12A, Q13A, and K20A agreed well with the remarkable changes of these TPN_Q_ mutants' binding affinities. In addition, the consistency between computational and experimental data was also observed for unimportant residue Arg7 and Trp15, whose substitutions led to minor decrease of TPN_Q_ toxin binding affinity. As for rKir1.1 channel, the two biggest ΔΔ*G*_binding_ values of F146A and F148A corresponded with their most significant mutagenesis effects on TPN_Q_ toxin binding. The replacements of residue Asp116, Arg118 and Thr119 with alanine caused moderate drop of TPN_Q_ binding affinity, which were also in accordance with the calculated ΔΔ*G*_binding_ values. In summary, the good confidence between the experimental and computational data indicated that the final TPN_Q_ toxin-rKir1.1 channel complex was a reasonable model, which could be used to explore the molecular mechanism of interaction between TPN_Q_ toxin and rKir1.1 channel through the structural analysis.

### Novel mechanism of TPN_Q_ toxin recognizing rKir1.1 channel

**Figure 4:**
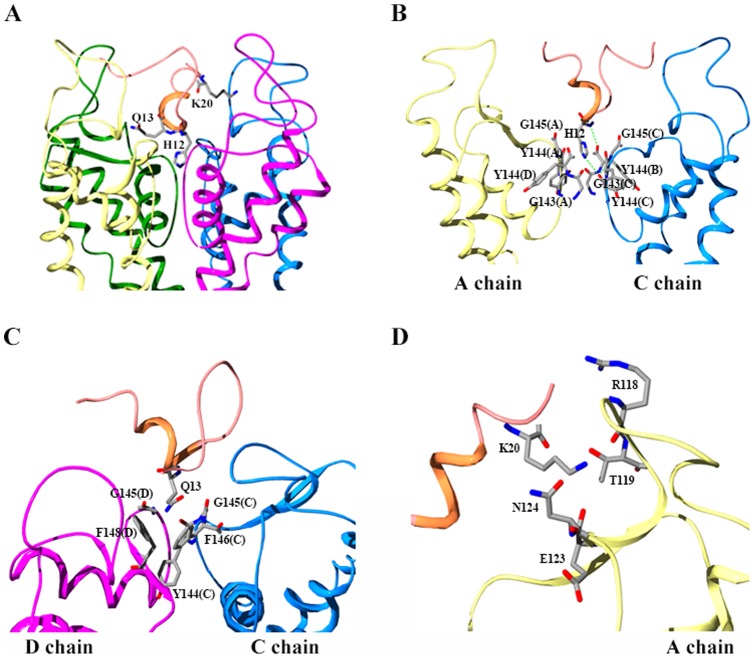
An overview of the TPN_Q_-rKir1.1 complex and interaction details of critical residues in TPN_Q_ toxin. (A) An overview of the final model of the TPN_Q_-rKir1.1 complex showing the critical His12, Gln13 and Lys20 residues of TPN_Q_ toxin. (B) Pore-blocking residue His12 mainly contacted conserved residues in the channel selectivity filter with two hydrogen bonds. (C) Gln13 residue mainly contacted Tyr144, Gly145, Phe146 residues in channel C chain, Gly145 and Phe148 residues in channel D chain. (D) Lys20 residue was surrounded by a polar “groove” formed by Arg118, Thr119, Glu123, and Asn124 residues in channel A chain.

Previous experiments demonstrated that three important His12, Gln13 and Lys20 residues seriously affected TPN_Q_ toxin binding affinity [14], which could be well elucidated in the TPN_Q_ toxin-rKir1.1 channel complex structure. As shown in Fig. 4A, TPN_Q_ toxin mainly adopted its α helical domain to recognize the vestibule of rKir1.1 channel, and this interaction mode was completely different from the known animal toxin-potassium channel interaction models. Different from the known pore-blocking Lysine residue in other animal toxins [3], [9], the pore-blocking residue was the most important His12 in TPN_Q_ toxin. As shown in Fig. 4B, His12 residue was surrounded by the channel conserved “GYG” motif within a contact distance of 4 Å, and it formed two hydrogen bonds with Gly143 in the channel C chain and Tyr144 in the channel B chain. These strong interactions between the toxin His12 residue and channel residues could well explain the most significant effect of His12 on TPN_Q_ toxin binding affinity [14]. The critical Gln13 residue was just adjacent to the pore-blocking His12, and located near the selectivity filter of rKir1.1 channel (Fig. 4C). Structural analysis indicated that toxin Gln13 residue contacted Tyr144, Gly145, Phe146 in channel C chain, Gly145 and Phe148 in channel D chain within a contact distance of 4 Å. These polar and non-polar interactions supported the important role of Gln13 in TPN_Q_ toxin binding capacity [14]. In addition, the TPN_Q_ toxin-rKir1.1 channel complex structure also rationalized the effect of the third important Lys20 residue on TPN_Q_ toxin function [14]. As shown in Fig. 4D, toxin Lys20 residue was surrounded by a polar “groove” formed by Arg118, Thr119, Glu123, and Asn124 in channel A chain within a contact distance of 4 Å. Together, these structural features of TPN_Q_ toxin-rKir1.1 channel interaction indicated that TPN_Q_ toxin used a novel mechanism to recognize rKir1.1 channel.

### Unique role of rKir1.1 channel vestibule in the toxin recognition

**Figure 5:**
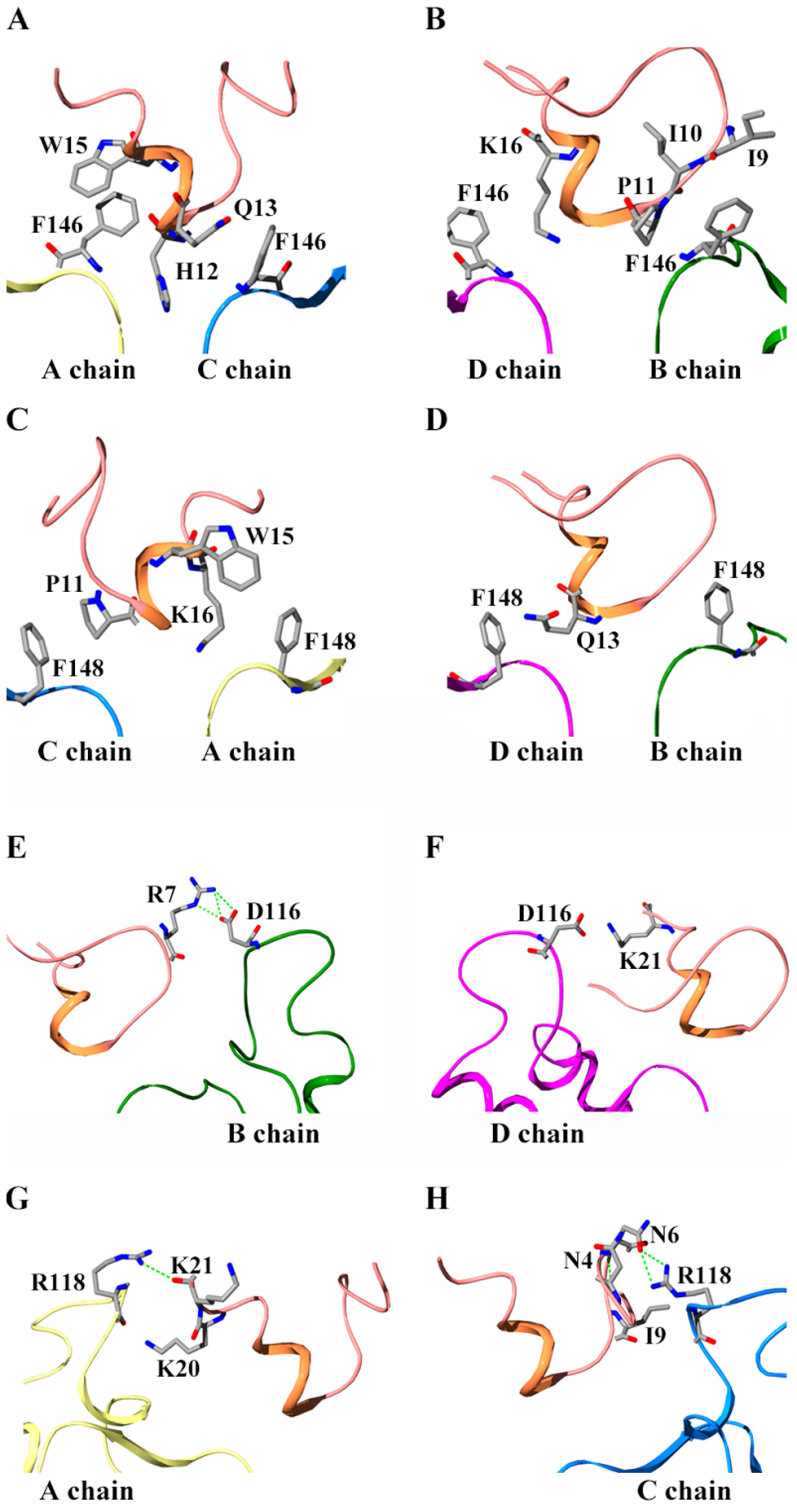
Interaction details of critical residues in rKir1.1 channel. (A and B) Four Phe146 residues formed hydrophobic protrusions, and contacted multiple TPN_Q_ residues including Ile9, Ile10, Pro11, His12, Gln13, Trp15 and Lys16 within a contact distance of 4 Å. (C and D) Four Phe148 residues formed hydrophobic protrusions, and contacted Pro11, Gln13, Trp15 and Lys16 residues in TPN_Q_ toxin. (E and F) Asp116 residues in channel B and D chains respectively formed electrostatic interactions with toxin Arg7 and Lys21 residues. (G and H) Arg118 residue in channel A chain interacted with toxin Lys20 and Lys21 residues, and Arg118 residue in channel C chain contacted toxin Asn4, Asn6 and Ile9 residues within a contact distance of 4 Å.

The experimental alanine scanning mutagenesis showed that the Phe146 and Phe148 residues near the selectivity filter and turret of rKir1.1 channel formed the binding site for TPN_Q_ toxin [14]. These functional features were found reasonable in the TPN_Q_ toxin-rKir1.1 channel complex structure. Similar to the orientation of Phe148 residue near the selectivity filter in cKir2.2 channel [15], four Phe146 residues, in the corresponding position of Phe148 residue in cKir2.2 channel, formed protrusions on the surface at the pore region of rKir1.1 channel (Fig. 5A and 5B). Within a contact distance of 4 Å, there were strong nonpolar interactions between four Phe146 residues and many toxin residues including Ile9, Ile10, Pro11, His12, Gln13, Trp15 and Lys16, which well explained the fact of rKir1.1-F146A mutant channel had 50-fold lower affinity for TPN_Q_ toxin [14]. Structural analysis indicated that four Phe148 residues also formed protrusions on the surface at the pore region of rKir1.1 channel, and they closely contacted Pro11, Gln13, Trp15 and Lys16 residues in TPN_Q_ toxin (Fig. 5C and 5D). These residue-residue interactions supported the important effect of Phe148 mutation on TPN_Q_ toxin binding [14]. Besides the pore region of rKir1.1 channel, the function of channel turrets was well elucidated according to the TPN_Q_ toxin-rKir1.1 channel complex structure. For example, alanine replacement of Asp116 and Arg118 moderately reduced channel affinity for TPN_Q_ toxin binding [14]. In accordance with this effect, Asp116 residues in channel B and D chains respectively formed strong electrostatic interactions with toxin Arg7 and Lys21 residues (Fig. 5E and 5F). Meanwhile, Arg118 residue in channel A chain interacted with toxin Lys20 and Lys21 residues, and Arg118 residue in channel C chain contacted toxin Asn4, Asn6 and Ile9 residues together with three hydrogen bonds (Fig. 5G and 5H). In summary, the vestibule of rKir1.1 channel played a unique role in the TPN_Q_ toxin recognition.

## Discussion

The molecular and structural diversities of animal toxins lead to the varieties of their interaction modes towards potassium channels at the structural level. Due to the huge challenge of experimental techniques, there is no animal toxin-potassium channel complex structure to be determined so far. However, the computational approaches have yielded many valuable structural insights into the diverse animal toxin-potassium channel interactions, and accelerated the drug development of animal toxins or their analogs targeted the potassium channels [3]–[7], [11], [12], [24], [25].

Recently, a TPN_Q_ toxin-human Kir1.1b channel complex model was predicted by the molecular docking and MD simulations [26]. In this work, the interaction between TPN_Q_ toxin and rKir1.1 channel was systematically investigated by molecular docking and MD simulations, and our complex model further confirmed the previous findings on TPN_Q_ toxin binding mode and the importance of His12 and Lys20 residues for toxin binding [26]. Interestingly, the TPN_Q_ toxin-Kir1.1 channel complex structures were different from the known animal toxin-potassium channel interactions, and this novel interaction mode would further highlight the diverse animal toxin-potassium channel interactions, and likely elucidate the relative insensitivity of rKir1.1 towards animal toxins.

### Diverse animal toxin-potassium channel interactions

In this work, TPN_Q_ toxin is a compact peptide of 21 residues, which forms a coil conformation in the N-terminal half and an α helical structure in its C-terminal portion. This structure is completely different from those of other kinds of potassium channel-blocking animal toxins [2]. Furthermore, TPN_Q_ toxin mainly adopted its helical domain as its channel-interacting surface together with His12 as the pore-blocking residue (Fig. 4A and 4B), which was also found in the predicted TPN_Q_ toxin-human Kir1.1 channel complex [26]. Previously, the inhibition potency of TPN_Q_ toxin towards rKir1.1 channel was found to be increased in the lower pH due to the presence of His12 residue [27]. As shown in Fig. 4A and 4B, toxin His12 located in the entrance of channel ion selectivity filter, and its protonation in the lower pH would be helpful for the polar interactions between toxin His12 and the channel conserved “GYG” motif. It was noticed that the mechanism of TPN_Q_ toxin recognizing rKir1.1 channel had not been observed in other kinds of animal toxins so far. Toxins from scorpions, snakes, cone snails etc. usually adopted lysine residue as the pore-blocking residue, which located in different second structure domains [3], [9], [28]. In addition, TPN_Q_ toxin had three important His12, Gln13 and Lys20 residues, which seemed not to have conserved functional dyad residues. Many animal toxins from scorpions, snakes, cone snails etc. possessed a conserved functional dyad, comprising a pore-blocking lysine residue near an important Tyr, Phe, or Leu residues [29]. Therefore, the novel binding mode of TPN_Q_ toxin further highlighted the diverse animal toxin-potassium channel interactions.

### Relative insensitivity of rKir1.1 channel towards animal toxins

The vestibules of potassium channels are the determinants responsible for animal toxin binding. These channel vestibules are composed of turret and pore region. So far, many classical animal toxins do not block rKir1.1 channel, and the TPN_Q_ toxin-rKir1.1 channel complex structure was helpful to elucidate the relative insensitivity of rKir1.1 channel towards animal toxins.

First, rKir1.1 channel had a unique turret structure which formed a narrow pore entryway for animal toxins. Different types of the potassium channels have diverse structure features in their turrets, such as the crystal structure of 10-residue turret in rKv1.2 channel [16], crystal structure of 20-residue turret in cKir2.2 channel [15], modeled structure of 19-residue turret in BK_Ca_ channel [6] and modeled structure of 42-residue turret in hERG channel [4]. According to the TPN_Q_ toxin-rKir1.1 channel complex structure, four 20-residue turrets gathered to constrict the pore entryway and form the binding site for TPN_Q_ toxin (Fig. 5E-5H). On the contrary, the similar long turrets of BK_Ca_ channel and much longer turrets of hERG channel kept far away from the bound scorpion toxins, and did not affect toxin binding [4], [6].

Second, three Phe146, Arg147 and Phe148 residues near the pore region of rKir1.1 channel also greatly contributed into the channel insensitivity towards animal toxins. As shown in Fig. 5A and 5B, four Phe146 residues created four significantly hydrophobic protrusions near the channel selectivity filter, and mainly produced hydrophobic interactions with the toxin residues. However, this special Phe146 residue in rKir1.1 channel is usually replaced by the conserved negatively charged Asp residue in the animal toxin-sensitive potassium channels, which formed dominant polar interactions with toxin residues [6], [9], [23]–[25], [28], [30]–[32]. Four Arg147 residues formed the positively charged potential patches in the pore region of rKir1.1 channel, which were expected unfavorable for the potent animal toxins with some basic residues in toxin binding interfaces [6], [9], [23], [32]. In addition, there were another significantly hydrophobic protrusions formed by four Phe148 residues (Fig. 5C and 5D), which never appeared in the corresponding position of Phe148 residue in the animal toxin-sensitive potassium channels [6], [9], [23], [28], [31], [32]. Importantly, the variable residue in the corresponding position of Phe148 residue in rKir1.1 channel was found critical for animal toxin binding in different potassium channels [33].

In summary, the TPN_Q_ toxin-rKir1.1 channel complex structure would be helpful to yield valuable insights into the unique role of specific vestibule structure in channel insensitivity towards classical animal toxins.

## Conclusions

The interaction between honey bee toxin TPN_Q_ and rKir1.1 channel was systematically investigated by the computational approaches. The segment-assembly homology modeling method was used to model a good starting rKir1.1 channel structure, which indicated the flexible conformation of channel turret. On the basis of the refined rKir1.1 channel structure, a reasonable TPN_Q_ toxin-rKir1.1 channel complex structure was obtained. In the novel interaction mode, TPN_Q_ toxin mainly adopted its helical domain as its channel-interacting surface together with His12 as pore-blocking residue. Moreover, TPN_Q_ toxin-rKir1.1 channel complex structure well elucidated the function of channel turrets and pore region for TPN_Q_ toxin recognition. The structural analysis indicated that four turrets of rKir1.1 channel gathered to form a narrow pore entryway, and Phe146 and Phe148 residues formed strong hydrophobic protrusions. These specific structure features of rKir1.1 channel vestibule likely restricted the binding of the classical animal toxins. Together, the TPN_Q_ toxin-rKir1.1 channel complex structure not only revealed their novel interaction mechanism, but also would highlight the diverse animal toxin-potassium channel interactions, and elucidate the relative insensitivity of rKir1.1 channel towards animal toxins.
